# On the Forensic Use of Y-Chromosome Polymorphisms

**DOI:** 10.3390/genes13050898

**Published:** 2022-05-17

**Authors:** Peter de Knijff

**Affiliations:** Department of Human Genetics, Leiden University Medical Center, P.O. Box 9600, 2300 RC Leiden, The Netherlands; knijff@lumc.nl

**Keywords:** Y-STR, Y-SNP, haplogroup, haplotype

## Abstract

Nowadays, the use of Y-chromosome polymorphisms forms an essential part of many forensic DNA investigations. However, this was not always the case. Only since 1992 have we seen that some forensic scientists started to have an interest in this chromosome. In this review, I will sketch a brief history focusing on the forensic use of Y-chromosome polymorphisms. Before describing the various applications of short-tandem repeats (STRs) and single nucleotide polymorphisms (SNPs) on the Y-chromosome, I will discuss a few often ignored aspects influencing proper use and interpretation of Y-chromosome information: (i) genotyping Y-SNPs and Y-STRs, (ii) Y-STR haplotypes shared identical by state (IBS) or identical by descent (IBD), and (iii) Y-haplotype database frequencies.

## 1. A Brief History of Y-Chromosome Polymorphisms

After the discovery of the first genetic polymorphisms on the human Y-chromosome in 1985 [1,2], it took the scientific community considerable time to appreciate the Y-chromosome for its true value. In 1993, it was—I presume, jokingly—suspected to associate with traits such as “*Ability to identify aircraft (DC10)*” and “*Preadolescent fascination with Arachnida & Reptilia (MOM-4U)*” [3]. In 1995, it was the *absence* of any polymorphism in a 729 bp. intronic fragment of the ZFY-gene, located close to the pseudo-autosomal region on Yp [4] among 38 males, which led to the reconstruction of “Adam”, the Y-chromosomal partner of “mitochondrial Eve” [5,6]. In their first review, also in 1995, describing the multidisciplinary use of Y-chromosome polymorphisms, Jobling and Tyler-Smith [7] list most of the Y-chromosome polymorphisms that were known at the time, including 19 loci that could, in principle, be genotyped by means of PCR. Especially relevant for their potential forensic genetic use was the discovery of the first short-tandem-repeat (STR) loci by Roewer et al. [8] (in 1992), and Mathias et al. [9] (in 1994). Also in 1992, Roewer and Epplen [10] were the first to use—amongst others—the Y-STR locus (27H39LR/DYS19) in the first Y-STR-related forensic case. In 1996, the so-called “minimal” set of Y-STR loci saw its first light [11]. Extensive details on this set of 8 Y-STR loci, in addition to four others, were provided in a number of back-to-back papers and reference tables published in the International Journal of Legal Medicine [12,13,14]. In the same year (1997), the first Y-STR mutation rate study on this set of loci was published [15]. The first cold-case use of Y-STRs was published in 1999 [16], the first in-depth global population comparison was published in 2001 [17], and the first forensic population dragnet application of Y-STRs was published in 2002 [18].

**Figure 1:**
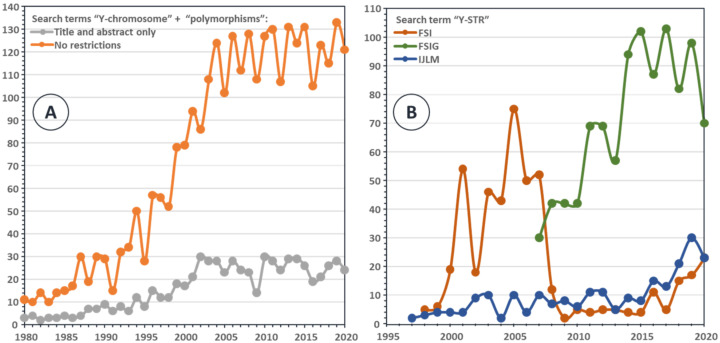
Bibliographic summary of published papers: (**A**) Result of a PubMed search in the years 1980–2020 with the terms “Y-chromosome” + “polymorphisms” either restricted to the title and abstract of the paper (grey) or without any restriction (orange). (**B**) Result of a PubMed search in the years 1995–2020 with the term “Y-STR” (without further restrictions) in the content of three forensically relevant journals, *Forensic Science International* (orange), *Forensic Science International: Genetics* (green; published since 2007), and *International Journal of Legal Medicine* (blue).

In the first years of their forensic use, Y-STRs were mainly genotyped by means of in-house designed singleplex and multiplex Y-STR PCR protocols. As a service to potential users, (multiplex) PCR designs—and protocols, allelic ladders, reference samples, and background information were distributed to over 150 laboratories worldwide between 1995 and 2002 [12,13,14]. The first commercial multiplex Y-STR kits (targeting five and six STRs, respectively) were issued by Reliagene in 2003 [19], soon followed by the Powerplex-Y (Promega) in 2005, enabling the simultaneous amplification of 12 Y-STR loci [20]. The importance of the availability of such kits is perhaps best illustrated by the rapid growth of published articles using Y-chromosome polymorphisms starting around 2000 (Figure 1). A literature search using the two terms “Y-chromosome” and “polymorphisms” (Figure 1A) in PubMed [21] clearly shows that, starting in 1995, there was rapid growth, until 2005, of published articles containing these two terms. Since 2005, there is no further rise in the number of articles published each year with these search terms. A more restricted search within three of the major forensic genetic journals with the search term “Y-STR” (Figure 1B) shows a similar trend.

Already in the first few years of the development of Y-chromosome polymorphisms, it was realized that a reference database with frequencies-information would be crucial. Exactly such a database, the YHRD [22], was released in 2000 and relevant details were published in 2001 [23]. To cite the authors of this publication: “*A long-term international project, initiated in 1994 to facilitate the forensic exploitation of human Y-chromosomal STR markers, has resulted in a database of European haplotype data (YHRD) that should be equally useful in forensic analysis and anthropological or archaeological research. The database is available to all interested users who are also invited to become collaborating contributors, thereby ensuring that the ongoing expansion of YHRD will benefit the forensic and scientific community*”.

Because a database without data makes no sense, it was also decided to coordinate the various research initiatives and share data and opinions, by means of a low-key and informal workshop: “1st Y-Chromosome User Workshop”, on 19–20 April 1996 in Berlin [22]. This workshop proved so popular that it grew into a more-or-less bi-annual meeting series, not only restricted to the Y-chromosome, but also embracing the aficionados of that other important forensically relevant lineage marker, the mitochondrial DNA, into their midst.

## 2. Y-STRs and Y-SNPs

For routine forensic or population genetic DNA research, one can use two distinctly different types of Y-chromosome polymorphisms, either on their own or combined: Y-chromosome STRs and single nucleotide polymorphisms on the Y-chromosome (Y-SNPs) (for detailed previous reviews on these see [7,13,24,25,26]. Especially in the early days of routine Y-chromosome research, only a limited number of Y-STRs could be genotyped efficiently by means of PCR [11,19,20]. This core set of Y-STRs consists of the tri-nucleotide repeat locus DYS392 and the tetra-nucleotide repeat loci DYS19, DYS385, DYS389-I, DYS389-II, DYS390, DYS391, and DYS393. Of these, DYS385 and DYS389 have some intrinsic complexities that are explained in detail elsewhere [27,28], but, in short, the full-repeat motif of DYS385 is present in two copies that are ~40 kb apart on the Y-chromosome, whereas DYS389, as a single locus, has four repeat-motifs within a ~400 bp fragment, of which three are known to be variable. This set of eight Y-STR-loci is present in all major Y-STR multiplex kits: PowerPlexY, Yfiler, PowerPlexY23, Argus-Y-28, GoldenEye, STRtyper-27, YfilerPlus, PathFinderPlus, AGCUY37, and YfilerPlatinum (information retrieved from [22]). In the latest version of YHRD, R67, there are 343,932 haplotypes consisting of these eight core loci available. A further three tetra-nucleotide loci, DYS437, DYS438, and DYS439, are also included in all of the above-listed Y-STR kits. Of special importance is the availability of a number of so-called rapidly mutating (RM) Y-STR loci. First described by Ballantyne et al. in 2012 [29], the inclusion of some of these RM-Y-STRs in Y-STR genotyping/sequencing kits is slowly gaining some momentum. With these RM-Y-STRs, one has a much better chance to be able to distinguish closely paternally related male relatives [29,30], which is obviously a major advantage in some forensic cases. Most of the currently available multiplex Y-STR CE kits allow the routine genotyping of 15–30 Y-STR loci [31,32,33,34]. This, however, will be changed with the introduction of massively parallel sequencing (MPS), enabling the sequencing-based genotyping of anything between several 10s up to several 100-eds of Y-STR loci simultaneously [35].

The screening and use of Y-SNPs have a much more complex past. Different research groups, using Y-SNP polymorphisms for population genetic purposes, used six different haplogroup nomenclatures and a plethora of different screening techniques [36,37,38,39,40,41,42,43]. Luckily, this Babylonian nomenclatural confusion was solved, following the mtDNA example, into the terminology we still use today [44,45]. In contrast to Y-STRs, for which CE-based multiplex PCR assays are routinely used, there is still no uniform, simple, sensitive, and commonly accepted Y-SNP screening method. Once again, this might change soon, however, as a number of proof-of-concept studies have shown that hundreds to thousands of Y-SNPs can be reliably genotyped [35,46]. In addition to these targeted approaches, hybridization capture followed by MPS techniques have been developed [47,48], and tools to efficiently extract Y-SNP information from whole-genome shotgun sequencing data sets have become available [49]. It is to be expected that these massive screening methods will be further developed rapidly.

### 2.1. Haplogroups and Haplotypes

Already in the first reports on Y-STRs and Y-SNPs [7], the two different terms haplogroup and haplotype were used, but not always clearly defined. Jobling et al. [14] were the first to discuss the differential use of these two terms, and in 2000, de Knijff [50] gave clear definitions: “*Distinct Y chromosomes, defined solely on the basis of UME* (i.e., unique mutation events such as SNPs and insertion/deletions) *character states, are designated as “haplogroups”. Distinct Y chromosomes identified by STRs are designated as “haplotypes”, and Y chromosomes that are defined by the combination of UMEs and STRs are called “lineages*”. Some would argue that in the current whole-genome-sequencing era the abundance of SNP- and STR information from individuals will blur this distinction. However, especially in a forensic diagnostics setting, where terms and conditions must be clearly defined and also possible to use in retrospect, I would still prefer the use of these clearly distinct terms.

### 2.2. Identical by State (IBS) and Identical by Descent (IBD)

**Figure 2:**
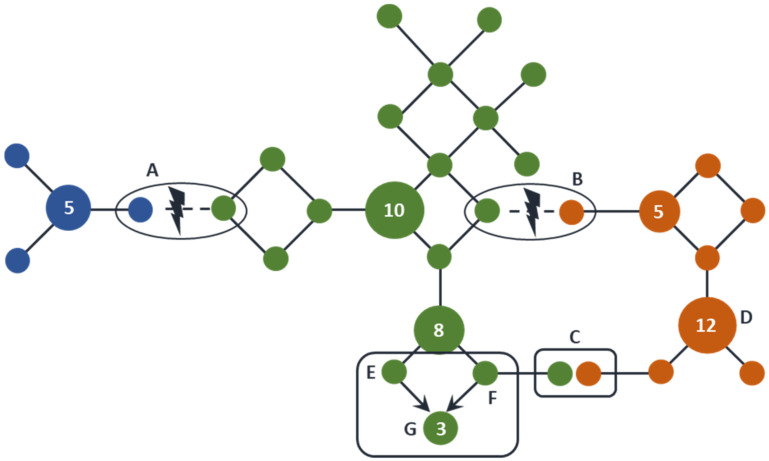
Y-STR haplotypes shared IBS and IBD. In this hypothetical set of 60 Y-chromosomes, a number of Y-STRs and Y-SNPs have been genotyped and connected into a network by single STR mutation steps. Each solid circle represents a unique Y-STR haplotype. The diameter of each circle represents the relative frequency of these haplotypes, further indicated with numbers inside. Unnumbered circles represent haplotypes that were observed once. The three different Y-SNP-defined haplogroups are shown in different colors. When, due to an SNP mutation, a new haplogroup arises (A and B) both the ancestral and derived Y-STR haplotypes are IBD, but at a different haplogroup. Similarly, also due to a series of independent and/or recurrent mutation events, two Y-STR haplotypes can be identical but occur on a different haplogroup background (C) or on a similar haplogroup background, but due to two parallel STR mutation events from different haplotypes (E and F), resulting in an identical haplotype (G). In both cases, they are shared IBS. Finally, males in a population can share Y-STR haplotypes IBD simply because they are (closely) related (e.g., D).

A frequently ignored or not always well-understood issue is the fact that two males, sharing an identical Y-STR haplotype, are not necessarily closely related per se. It has been demonstrated by many previously published (deep-rooting) pedigree studies that males sharing a most recent common paternal ancestor (MRCPA) at least up to 20 generations ago can have an identical Y-STR haplotype by descent (i.e., IBD) and also display the same Y-haplogroup (Figure 2). Males can also share identical Y-haplotypes at an identical Y-haplogroup background or at a different Y-haplogroup background purely by chance, i.e., they share these Y-haplotypes by state (IBS).

For Y-STR haplotype sharing IBS at a different Y-SNP (sub)haplogroup, there is empirical evidence [51,52,53], and it is likely to occur more frequently, especially when the haplotype consists of relatively few (<10) STR loci. Note, however, that even with haplotypes of 17 Y-STR loci, IBS was demonstrated [52]. Two haplotypes can also appear to be identical for another reason: repeat homoplasy. It was already reported in 1999 [54] that the total repeat count of a single STR allele can be identical, but with different underlying repeat sequences. With the recent increasing use of MPS instead of capillary electrophoresis (CE), this phenomenon has been reported to occur frequently, especially among the more complex compound autosomal and Y-STR loci [55,56,57]. Obviously, the combined effect of this is the simple notion that identical Y-STR haplotypes, especially those based on CE and consisting of less than 20 loci, can come from different unrelated “sources”.

### 2.3. Database Frequencies

**Figure 3:**
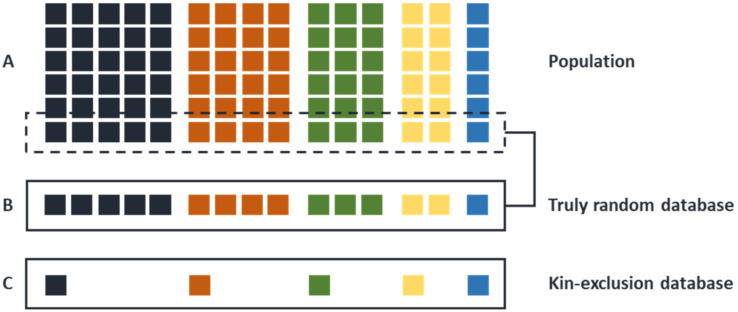
The difference between a random DNA database and a kin-selected DNA database. If we want to create a reference database of allele frequencies for autosomal STR loci in a human population (**A**), it was common practice to avoid the inclusion of biological relatives in such a population sample, i.e., an unknown number of reference databases are based on kin-exclusion (**C**). If the same DNA samples are subsequently used for the creation of a Y-STR reference database, such a kin-exclusion must, per definition, lead to the under sampling of large Y-kin groups. In the ideal case, as recently recommended [63], Y-STR reference databases should be based on truly sampling random individuals from a population irrespective of a possible biological relationship among them (**B**).

As already touched upon above, already at a very early stage of developing and introducing Y-chromosome polymorphisms in the forensic research realm, it seemed crucial to initiate a database with free access that would serve the need for population frequencies of Y-STR haplotypes. The first version of this database, the YHRD [22], was released in 2000 [23], and has since been extended and updated regularly [58,59]. Moreover, recommendations concerning how to estimate and report haplotype frequencies have also been published [60]. However, none of these studies deal with the most relevant aspect: do the database frequencies truly represent those from the various populations at large. Actually, as far as I am aware, no empirical studies into this crucial aspect have been published with respect to Y-STRs (nor for autosomal STRs); although, Andersen and Balding [61,62] discussed this aspect in some detail and provided a simulation-based solution. From a purely philosophical point of view, one could reason that many of our Y-STR population databases will, per definition, underestimate the frequencies of more common Y-STR haplotypes, and (slightly) overestimate the rare ones (explained in Figure 3). This is because many DNA sample sets were originally collected to estimate the allele frequencies of autosomal STR loci. From such collections close biological relationships were excluded because it was assumed that this would lead to inflated allele frequency estimates. However, if this were true, those kin-selected DNA sample sets would not truly represent the relevant Y-chromosome diversity pattern of the population at large, because from the more frequently occurring paternally related Y-STR haplotypes in that population only one (if at all) would be sampled. Recently [63], the latest recommendations with respect to reporting Y-STR haplotype frequencies were published. Depending on the relevant jurisdiction, one has a number of different reporting options, each of which primarily informs about the relative frequency of a relevant Y-STR haplotype in a given (meta)population. Irrespective of the method used, one should always very clearly inform those for which the report was written that any Y-STR haplotype is very likely never unique and that there will always be an unknown number of (related or not) males carrying a similar Y-STR haplotype. This is in marked contrast to the current autosomal STR profiles, which are, monozygotic twins excepted, almost always individual specific. Obviously, all of the above does not diminish the relative evidential value of Y-STR haplotype frequencies, it only demands a more careful phrasing of the conclusions. Furthermore, and equally important to finding a possible match, Y-chromosomal polymorphisms can also be used to exclude possible suspects as a potential donor of cell material present in a crime-scene sample.

With respect to the availability of Y-SNP-defined Y-haplogroup frequencies, I can be very short. Apart from the semi-quantitative haplogroup information included in the YHRD, I am not aware of any other freely available database one could use to search for Y-SNP-defined haplogroup frequencies. The one database of which I am aware that can be used to search for Y-haplogoups is the aYChr-DB [64], based on 1400 ancient DNA samples, and is hardly relevant for modern-day forensic use. In my lab, we use an in-house database with the Y-haplogroups of ~50,000 globally dispersed males, and I assume many other labs will have something similar. Based on a recent literature search (unpublished), it would be fairly simple to gather the Y-haplogroups of varying complexity of at least 250,000 globally distributed males, giving plenty of opportunities for an ambitious new database builder, or a new and better accessible branch of the YHRD.

## 3. The Various Applications of Y-STRs and Y-SNPs

One of the earliest uses of genetic variation in the human Y-chromosome was the so-called “gender identification” of female athletes. This test, based on the intron-length variation between AMELX and AMELY, is, apart from ethical issues [65], not without technical problems [66,67]. Basically, this test, at most, indicates the presence of an X-chromosome and/or a Y-chromosome in the DNA sample that is tested. This, however, as some of us have already known for a long time, is not the same as the identification of the sex (or gender) of the donor of the DNA sample [68,69]. Nowadays, many multiplex autosomal PCR kits not only include AMELOGENIN as a potential “gender-marker”, but also include various other Y-chromosome loci for this purpose (see [70] and references therein).

Once the use of autosomal STR loci for forensic use became commonly accepted and used [71], it soon became apparent that in crime-scene samples with a mixed origin, minor components below a contribution of 10–20% of the total DNA amount were difficult or impossible to detect because of allelic dropout [72,73]. This is especially relevant for sexual assault samples that often have a (very) minor male component and a high female background [72]. For such challenging samples, it was reasoned that the use of Y-STRs would be ideally suited as they would not suffer from preferential amplification [74]. Roewer and Epplen [10] were the first to show that this assumption was correct, and a number of studies using male/female mixtures showed that a minor male component as low as 1:3000 or less still rendered Y-STR profiles [74,75,76,77]. As became serendipitously clear in one notoriously difficult GENAP proficiency test (a few participating laboratories did discover and report Y-STR results where none were expected by the organizers), the additional power of the use of Y-STRs was demonstrated to be effective even among vasectomized males [78]. In his recent reviews, Roewer [79,80], explains and summarizes many forensic applications of Y-STRs in great detail. Instead of repeating them here, I thoroughly recommended these reviews for those who want to learn more. I will discuss only two approaches, forensic dragnets and biogeographical ancestry (BGA) reconstruction, in some detail below.

### 3.1. Biogeographic Ancestry Reconstruction Using Y-Chromosome Polymorphisms

**Figure 4:**
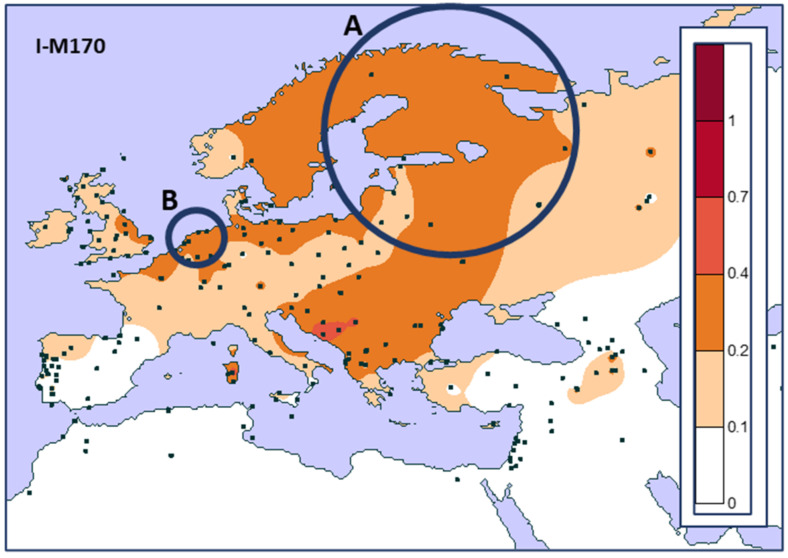
Prediction surface map of haplogroup I (M170) based on the frequencies from 144 sampling locations (shown as solid black squares) encompassing 2980 males. (**A**) Finland and neigbouring regions, see main text. (**B**) The Netherlands, see main text. The spatial patterns were created using ordinary Kriging interpolation implemented in Surfer software (version 7, Golden Software Inc., Golden, CO, USA) and shown with a 10% gradient step.

**Figure 5:**
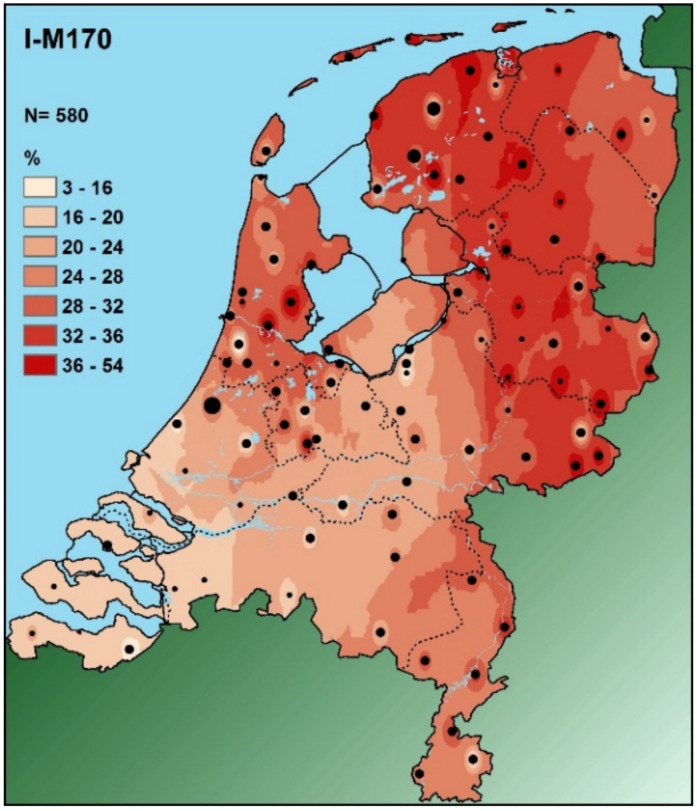
Prediction surface map of haplogroup I (M170) based on the frequencies from 99 Dutch locations (shown as solid black circles) encompassing 2085 males [83]. The spatial patterns were created using ordinary Kriging interpolation implemented in ArcGIS version 10.2 with the Spatial Analyst extension.

Since the use of autosomal polymorphic loci for the purpose of biogeographic ancestry (BGA) inference has been more than adequately described by Tvedebrink [81], I will here only focus on the use of the Y-chromosome polymorphisms for this purpose. Soon after the first (global) population-based genetic screenings with sets of Y-SNPs were published [38,39,40,41], it became clear that many of the Y-haplogroups showed a limited geographical distribution, which makes them a potentially very useful tool for BGA inference. In their very detailed review paper, Underhill and Kivisild [82] write in the legend to their Figure 1: “*Clusters or clines of genetic diversity? Genetic structure of human populations could well be compatible with both concepts as clines would not be detected if there was no genetic patterning*”. In other words, as is now obvious from many different global genetic surveys, geographically widely separated human populations can display a fair amount of discrete genetic profiles. However, if one were able to sample all the populations between them, it would rarely be possible to draw the line that clearly marks the border of their genetic distinction. It is for this reason that the degree of representativeness (and reliability) of any BGA inference strongly depends on the quality of the reference database used for this purpose. As a consequence, any BGA inference without any clear information concerning the reference database(s) is at least suspicious. This is perhaps best illustrated by an example (see Figure 4 and Figure 5).

Both maps (Figure 4 and Figure 5) show the geographical distribution of relative frequencies of the Y-haplogroup I (M170) in Europe and the Netherlands, respectively. Maps such as these are frequently used in order to support the use of Y-haplogroups for BGA inference. As such, they provide valuable information in a seemingly clear way. However, such maps should be used with caution. Methods such as Kriging (but there are also other interpolation methods, see e.g., [84,85]), will always fill in the empty spaces between the various sampling locations, depending on the grid dimensions used. Moreover, unless specifically disenabled, Kriging (and other methods) will also interpolate over stretches of water such as the Mediterranean Sea or unpassable mountain ridges such as most of the Himalayas. In Figure 4, the region marked with A is a vast area with a very limited number of data points that gives the impression of a uniform Y-haplogroup I (M170) frequency of 20–40% in Finland, despite the complete absence of any empirical estimate from that country in this data set. In order to better infer the reliability of these “filled-in” unsampled regions, Bayesian geostatistical modeling methods have been developed [85] and used [86]. It is for this purpose that in other applications of a similar method, not only is the estimated landscape with sampling locations shown, but also a separate map showing the relative reliability of these estimates, which is especially relevant for unsampled regions [86]. To my knowledge, such a fully informative approach has never been developed and made available for BGA-inference purposes. What Figure 4 also shows is the importance of *always* showing the sampling locations. This at least enables the user to appreciate the coverage of data points, based on which one could infer that the Finnish estimate in this map is not as reliable as it appears. The region marked with B in Figure 4 reflects the Netherlands, one of the most densely sampled—in terms of forensically relevant genetic loci—European regions. From Figure 4, based on five Dutch sampling points, one could get the impression that there is a uniform Dutch Y-haplogroup I (M170) frequency distribution. However, from Figure 5, based on 99 sampling locations, it is clear that there is no uniform Dutch Y-haplogroup I (M170) distribution [83]. In contrast, there is a very clear decreasing frequency gradient from NE to SW. From the above, one should conclude that, yes, there are marked globally distributed Y-haplogroup frequency differences, but these are rarely simple and should always be treated and used with care. The example shown here also very clearly illustrates that, as already stated above, it is *the absence* of reference data that will ultimately define the accuracy of any Y-haplogroup (or any other marker set)-based BGA inference. It is for this reason that I prefer not to use an LR in order to support such a BGA inference, simply because this LR is unable to inform about the absence of relevant reference data. Instead, I advocate the use of a verbal statement explaining the relative frequencies and their caveats, supported by maps such as those in Figure 4 and Figure 5. Since in most cases, a forensically relevant BGA inference is used in the investigative phase and not used to support a conviction (in court), such a verbal statement would probably be acceptable under most jurisdictions.

The above is based on Y-SNPs only. Obviously, Y-STRs can also be used (either on their own or combined with Y-SNPs) for BGA inferences. A very good and elegant example of this was published by Diaz-Lacava et al. [87]. Geostatistical clustering of 33,010 seven loci (DYS19_389I_389II_390_391_392_393) haplotypes, collected at 249 sites in Europe, Western Asia, and North Africa and present in the YHRD (release 29 from June 12, 2009), they identified a series of haplotype clusters, each of which showed a distinct geographical distribution. A number of other approaches also revealed Y-STR haplotypes with specific geographical distributions. One of these, the so-called Atlantic Modal Haplotype [88] (14-13-29-24-11-13-13 for DYS19_389I_389II_390_391_392_393), is one of the most frequent haplogroup R1b Y-STR haplotypes on the western fringes of the European continent. Perhaps the most famous (or notorious) Y-STR haplotype was coined the Genghis Khan haplotype because of its very specific high frequency central Asian distribution [89]. Later, more of such specific Y-haplotype clusters were discovered and described [90,91,92]. In addition, at a smaller geographical scale, it is not that hard to find groups of males, often carrying an identical or very similar surname carrying (near)identical Y-STR haplotypes. King and Jobling [93] give a very detailed review on this topic, which is also highly relevant for the next topic of this review.

### 3.2. Y-Chromosome-Based Dragnets

Males who are patrilineal related will share (near) identical Y-chromosomes. It is therefore not surprising that the convenient direct link between specific Y-chromosome lineages and (male) surnames has been studied in detail [15,55,94,95,96,97]. Obviously, the importance of surname clusters has been known (indirectly) for a much longer time. Already in 1965, Crow and Mange [98] were among the first to realize the usefulness of the relation between isonomy (i.e., sharing of surnames) and inbreeding to study population structure. As Manni [99] states “*In societies that use a patrilineal transmission of surnames, family names simulate neutral alleles of a gene transmitted only through the Y-chromosome, and therefore satisfy the expectations of the neutral theory of molecular evolution, which is entirely described by random genetic drift, mutation, and migration*”. Hence, a detailed knowledge of local surnames can therefore be, already, on its own, a powerful (forensic) research tool [100]. This makes it possible, using a combination of genealogical research and Y-STR haplotyping, to identify a suspect of a serious crime using the Y-STR information of one or more (distant) paternal relatives.

In those forensic cases where we have enough relevant criminalistic evidence, detailed DNA profiles, but no suspect, a whole set of new forensic genetic/genomic methods have become available to assist the police in finetuning or narrowing down their search for a suspect [101,102]. However, if all this fails, there is one resort option: to invoke a so-called Y-chromosome dragnet (of course assuming the suspect is a male). The underlying principle is simple, it requires the availability of an as detailed as possible Y-STR/Y-SNP profile of the male perpetrator of a crime. Under the assumption that in the relevant population, there will be (many) more paternally related males with (nearly) similar Y-lineages, it could be possible to indirectly identify the perpetrator. If it is possible to screen, on a voluntary basis, an as large as logistically and legally possible group of males, it is hoped that at least one of these paternal relatives participates. His Y-profile will match that of the male perpetrator, but his autosomal STR-profile will be different. Based on the matching participant’s surname, genealogical studies will result in a detailed pedigree (sometimes even linking to different surname branches) making it possible to identify more possible participants and possibly also the perpetrator. Police investigations can, once the dragnet is completed, delete all false-positive Y-matches, and focus their search on those who were not invited and those who did not participate or declined to participate. The first successful example of this approach was used in Poland [18]. In the first sample of 420 (out of a possible 12,000) volunteers, one male with a matching 9-locus Y-STR haplotype was found, who turned out to be a full brother of the perpetrator, who was apprehended a few days after the match.

My laboratory has been involved in a number of similar Dutch dragnet cases. Details of the first, involving a young girl, Marianne Vaatstra (MV), who was murdered on the night of 30 April and 1 May 1999 in a rural area in the north of the Netherlands, were published (by others) elsewhere [26,103]. Details of a second, also involving a young girl, Milica van Doorn (MvD), who was murdered on the night of 7–8 June 1992 in Zaandam, just north of Amsterdam, are not fully released (the case is still on appeal), but some relevant aspects were discussed elsewhere (by others) [104]. What both cases have in common is the fact that the dragnets were preceded by BGA analyses. In the case of the first (MV), this was initially solely based on a 9-locus Y-STR search, in 2000, using the first version of YHRD, and a number of additional datasets (published but not present in the YHRD). This Y-based BGA suggested that the perpetrator could be of north-western European origin. Furthermore, it showed that it was very unlikely that the perpetrator would be from Iraqi–Afghani–Kurdish origins, as was commonly assumed by the public at large (because, close to the crime scene, there was a political asylum where immigrants from this geographical region were housed). In the second case (MvD), the Y-based BGA suggested that the suspect could be of east-Turkish origin; although, neighboring regions could not be excluded because reference profiles from these regions were not available. In the first case (MV), a local Y-dragnet, involving possibly 7600 males, saw 87% of them participating, and, already, in one of the first batches that was processed, a full Y-STR match was observed. Since the autosomal STR profile did not match, it provided the first crucial link to the suspect via his pedigree (branches of which showed different surnames). In the end, the suspect himself also volunteered, and was identified as such. In the second case (MvD), after a very carefully prepared operation, a limited number (<200) of males of Turkish descent were invited to participate on a voluntary basis. A brother of the suspect participated, which lead to the identification of the suspect, who had declined to participate voluntarily.

Despite the apparent success as a powerful investigative tool, Y-BGA approaches have many societal and ethical implications (see [104] for a critical discussion on this), and the decision to roll out a dragnet should not be made lightly. In all the Dutch cases in which my lab was involved, a special multidisciplinary team, not only consisting of police officials (in various capacities), but also DNA experts from different institutes, social science experts, public relation experts, and genealogical experts, were set up in order to prepare and guide the dragnet. From my own Dutch experience, I can conclude that, if used with care and performed well-prepared, a Y-based dragnet can be a very powerful forensic research tool. However, one has to be prepared for a sometimes-massive amount of work and unexpected public-relations aspects.

## 4. Conclusions

I hope that from the above it will become clear that, in contrast to common belief, the use of Y-chromosome polymorphic markers in a forensic context is not for the faint-hearted. Nothing is as it seems, and, in contrast to an autosomal STR profile, Y-STR profiles (haplotypes) will—rare cases excepted—not be individual specific. There will always be an unknown number of males with a similar profile. As a consequence, a Y-haplotype frequency (no matter how it is estimated) has a different evidential value. Nevertheless, the use of Y-chromosome polymorphisms can be a very powerful forensic research tool, if used and interpreted with care. Especially as an exclusion tool in complex male–female crime-scene sample mixtures, it has proven to be invaluable. Furthermore, its ideal suitability as a dragnet facilitator has demonstrated its usefulness. As one of the initiators of the use of Y-chromosome STRs in forensics, I strongly support the recommendations as outlined in the various ISFG publications on this topic [63]. However, as an independent scientist, I sometimes have slightly different opinions. These, I have tried to explain above.
